# Degree of tinnitus improvement with stapes surgery – a review^☆^

**DOI:** 10.1016/j.bjorl.2017.12.005

**Published:** 2018-01-05

**Authors:** Aliciane Mota G. Cavalcante, Isabella Monteiro de Castro Silva, Bianca Jessica Neves, Carlos Augusto Oliveira, Fayez Bahmad Jr

**Affiliations:** a Universidade de Brasília (UnB), Ciências Médicas, Brasília, DF, Brazil; b Universidade de Brasília (UnB), Escola de Pós-Graduação, Brasília, DF, Brazil; c Harvard Medical School, Otology and Neurotology, Boston, United States; d Universidade de Brasília (UnB), Faculdade de Ciências da Saúde, Brasília, DF, Brazil

**Keywords:** Otosclerosis, Tinnitus, Stapes surgery, Otosclerose, Zumbido, Estapedectomia

## Abstract

**Introduction:**

Otospongiosis is temporal bone osteodystrophy, characterized by disordered bone resorption and neoformation in genetically predisposed individuals. Clinically, otospongiosis is characterized by progressive conductive and/or mixed hearing loss and by tinnitus.

**Objective:**

A review of the last two decades of publications that report the degree of tinnitus improvement with stapes surgery.

**Methods:**

125 articles published in the last 20 years mentioning the relationship between otosclerosis and tinnitus. Literature has always shown that the hearing improvement after stapes surgery was the main result sought and found. However, recent articles has reinforced the need for surgery for the tinnitus improvement. The ideal time to assess tinnitus through different scales is in the sixth month post-operative. The estimated average hearing improvement is 93% and tinnitus is 85.52%.

**Results:**

Summaries of 12 articles were reviewed which fulfilled the search criteria of the survey, and 8 studies were included in the study according the selection criteria. This studies investigating the degree of tinnitus improvement with stapes surgery, using different scales as: tinnitus functional index, visual analog scale, tinnitus functional index and visual analog scale, visual analog scale and “questionnaire asking about tinnitus”, Newman's method and Tinnitus Score Advocated by the Japan Audiological Society. The total of the samples of the evaluated articles was of 254 participants.

**Conclusion:**

We conclude that stapes surgery is effective for the treatment of tinnitus (average improvement is 85.52%), and hearing loss (average improvement is 93%). When deciding about the surgical indication in patients with otosclerosis, the presence and level tinnitus should be considered as well as the level of hearing.

## Resumo

**Introdução:**

A otosclerose é uma osteodistrofia do osso temporal, caracterizada pela reabsorção e neoformação óssea desordenadas em indivíduos geneticamente predispostos. Clinicamente, a otosclerose é caracterizada por perda auditiva progressiva condutiva e/ou mista e por zumbido.

**Objetivo:**

Uma revisão das últimas duas décadas de publicações que relatam o grau de melhora do zumbido com a estapedectomia

**Método:**

Foram analisados 125 artigos publicados nos últimos 20 anos que mencionavam a relação entre otosclerose e zumbido. A literatura sempre mostrou a melhoria auditiva como principal objetivo e resultado da estapedectomia. No entanto, artigos recentes reforçaram a necessidade de cirurgia para a melhoria do zumbido. O momento ideal para avaliar o zumbido através de diferentes escalas é no sexto mês pós-operatório. A melhoria auditiva média estimada é de 93% e a do zumbido, de 85,52%.

**Resultados:**

Foram revisados resumos de 12 artigos que preencheram os critérios de pesquisa, sendo incluídos no estudo 8 artigos de acordo com os critérios de seleção. Este estudo investiga o grau de melhora do zumbido com a estapedectomia, utilizando diferentes escalas: *tinnitus functional index*, escala visual analógica, *tinnitus functional index* e escala visual analógica, escala visual analógica e “questionário sobre o zumbido”, método de Newman e *o Tinnitus Score Advocated*, da Sociedade Audiológica do Japão (*Japan Audiological Society*). O total das amostras dos artigos avaliados foi de 254 participantes.

**Conclusão:**

Concluímos que a estapedectomia é bastante eficaz no tratamento do zumbido (melhora média de 85,52%) e perda auditiva (melhora média de 93%). Ao decidir sobre a indicação cirúrgica em pacientes com otosclerose, a presença e o nível de zumbido devem ser considerados, assim como o nível de audição.

## Introduction

Otospongiosis is temporal bone osteodystrophy, characterized by disordered bone resorption and neoformation in genetically predisposed individuals. All the otic capsule may be involved, although the area close to the fissula ante fenestram (anterior to the oval window) is the most commonly affected site.

Clinically, otospongiosis is characterized by progressive conductive and/or mixed hearing loss and by tinnitus Sensorineural hearing loss, aural fullness and vertigo may eventually occur.

Tinnitus is an abnormal sound sensation that some patients with hearing loss experience. Patients with otosclerosis may experience variable degrees of tinnitus associated with their hearing loss. Gristwood et al.^1^ reported that 65% of patients with hearing loss due to otosclerosis have tinnitus based on a review of 1014 consecutive cases of clinical otosclerosis.

Then Deuyer et al. reported that tinnitus prevalence is estimated to be 65–85%.^2^ Previous studies have indicated that tinnitus does decrease when hearing improves after stapedectomy.^2^ Several studies have been talking about the high prevalence of tinnitus and the degree of discomfort in patients with otosclerosis and improvement after surgery. However, only a few previous studies in general have delineated the time frame of tinnitus improvement or quantified the improvement using a validated tinnitus instrument in a prospective fashion. The objective of this systematic review is to evaluate the result of publications that report the degree of tinnitus improvement with stapes surgery, with emphasis on the type of method used and the evaluation period.

## Methods

Searches were conducted in the databases PubMed, using the extracted descriptors of Medical Subject Headings (MeSH) that characterized the theme: otosclerosis AND stapes surgery OR stapedotomy AND tinnitus.

The inclusion criteria of the studies were: articles in English; published in the last 20 years; prospective study and clinical studies in adults with emphasis on the otosclerosis, stapes surgery and scales to measure the degree of tinnitus improvement. Retrospective studies were excluded.

## Results

**Figure 1:**
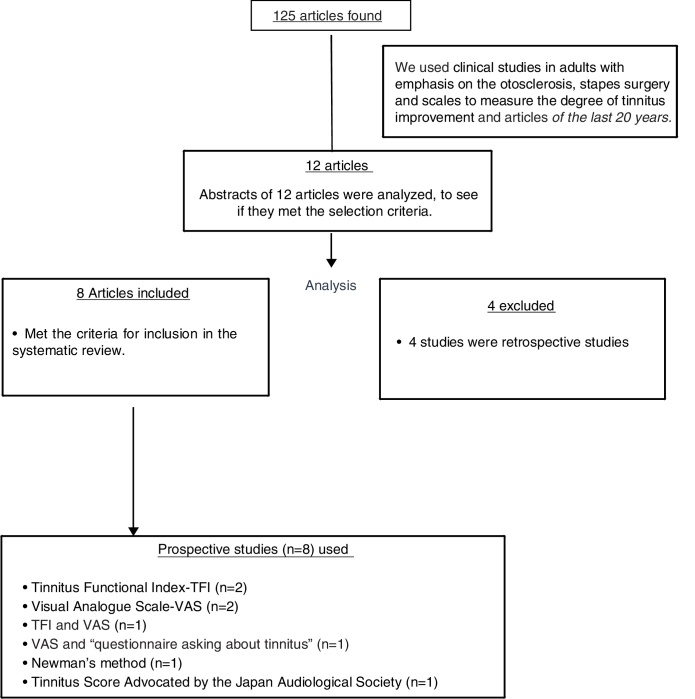
Diagrama PRISMA showing the selection and type of scale used in the review.

Summaries of 12 articles were reviewed which fulfilled the inclusion criteria of the survey, and 8 studies were included in the study according the inclusion criteria. Fig. 1 shows the flow diagram for inclusion.

### Characteristics of studies

This review found eight studies investigating the degree of tinnitus improvement with stapes surgery, using different scales. The stapes surgery were stapedectomy and stapedotomy. The articles used different scales as: Tinnitus Functional Index-TFI, Visual Analog Scale-VAS, TFI and VAS, VAS and “questionnaire asking about tinnitus”, Newman's method and Tinnitus Score Advocated by the Japan Audiological Society.

In the 1st month of postoperative evaluation, the results varied between 75% and 88% improvement in tinnitus. In the 6th month, between 85% and 88.3%.

Sakai et al. did not mention the evaluation period. In this article, the degree of improvement was 68%.

Sanchez et al. reported that the improvement around the 3rd month was of 95.7%, being the period of greatest degree observed among all the articles.

In studies in which the evaluation was done between 4 and 10 months; 4 and 14 months and 14 and 48 months, the degree of tinnitus improvement varied from 90 to 91%.

The total of the samples of the evaluated articles was of 254 participants (Table 1).

**Table 1:** Articles, type of Scales, evaluation time post-operative and results.

Title	Author/year of publication	Type of Scale	Sample size	Evaluation time post operative	Degree of tinnitus improvement
Stapedectomy Effects on Tinnitus: Relationship of Change in Loudness to Change in Severity^2^	Dewyer et al., 2015	TFI and VAS	35	1 and 6 months	1 month-75% 6 months-88%
Tinnitus modulation by stapedectomy^3^	Chang et al., 2014	TFI	16	1 and 6 months	1 month-88% 6 months-85%
Characteristics and postoperative course of tinnitus in otosclerosis^4^	Ayache et al., Earally, Elbaz, 2003	TFI	62	1 and 6 months	1 month-83.4% 6 months-88.3%
Outcome of stapes surgery for tinnitus recovery in otosclerosis^5^	Rajati, Poursadegh, Bakhshaee, Abbasi, Shahab, 2012	Newman's method	29	1 month	82.8%
The effect of stapedotomy on tinnitus in patints with otospongiosis^6^	Sanchez, Bento, Lima, Marcondes, 2005	VAS	23	3 months	95.7%
Long-Term Follow-Up of Tinnitus in Patients with Otosclerosis After Stapes Surgery^7^	Sobrinho, Oliveira, Venosa, 2004	Questionnaire asking about tinnitus, VAS	48	4–14 months; 14–48 months	4–14 months-91%; 14–48 months-91%
How does stapes surgery influence severe disabling tinnitus in otosclerosis patients?^8^	Oliveira, 2007	VAS	19*	4–10 months	4–10 months-90%
The effect on tinnitus of stapes surgery for otosclerosis^9^	Sakai, Sato, Iida, Ogata, Ishida, 1995	Tinnitus score advocated by the Japan Audiological Society	22	No mentioned	68%

## Discussion

Although tinnitus is often related to otosclerosis, it has been infrequently discussed in the literature. However, it represents a major source of discomfort for a few patients, who are often inquisitive about the course of this symptom.^2^

No postoperative tinnitus was observed in patients who were free of tinnitus preoperatively, but this factor did not seem to be statistically significant as a predictive indicator of the course of tinnitus. This finding was also noted by Kersley and Gray,^10^ but Del Bo et al.^11^ mentioned that tinnitus occurred later after surgery in 7% of patients who were free of tinnitus in the immediate postoperative period.

Shea^12^ and Causse and Vincent^13^ tried to correlate pitch of preoperative tinnitus in otosclerosis patients and decrease of this symptom after stapes surgery. Both stated that only low-tone tinnitus is affected by stapes surgery. Causse and Vincent indicated that this kind of tinnitus is related to the elasticity of the oval-window mechanism, which is corrected by stapes surgery.

In a temporal-bone study searching for a pathological correlate for tinnitus, Oliveira and Schuknecht^14^ found endolymphatic hydrops in 18% of the bones studied, normal histopathology in 11%, and otosclerosis in 11%. These were the major histopathological diagnoses found in tinnitus patients. If we consider that tinnitus starts with a biochemical alteration in the inner-ear fluids, which in the beginning will not be detectable by light miscroscopy but later is seen as endolymphatic hydrops, and that otosclerotic focuses in the cochlea provoke these biochemical changes in endolymph and perilymph, these major histopathological diagnosis found in temporal bones of tinnitus patients were tie together. If the foregoing explanation is true, the only way in which stapes surgery can influence tinnitus in otosclerosis patients is by changing the conductive part of the equation.

Again, Oliveira and Schuknecht^14^ found better preservation of sensory and neural structures in patients with tinnitus than in patients with the same histopathological diagnosis but without tinnitus. Possibly, tinnitus is a very early sign of cochlear lesion and tends to decrease as the lesion worsens. Of course, the ideas discussed in the preceding paragraphs are far from being proved, but we believe that they comprise an interesting hypothesis to be investigated.

## Conclusion

This review of 254 cases of otosclerosis showed through different scales and in different moments that stapes surgery was valuable in the improvement of tinnitus, which was observed in 85.52% of patients with preoperative tinnitus.

The primary indication for stapes surgery is to improve hearing.^4^

So, when deciding about the surgical indication in patients with otosclerosis, the presence and level of tinnitus should be considered as well as the level of hearing, as we have concluded that the stapes surgery can also alleviate tinnitus in most otosclerotic patients.

## Conflicts of interest

The authors declare no conflicts of interest.
